# Effect of crude plant extracts from some Oaxacan flora on two deleterious fungal phytopathogens and extract compatibility with a biofertilizer strain

**DOI:** 10.3389/fmicb.2014.00383

**Published:** 2014-08-05

**Authors:** Karla I. Lira-De León, Marco V. Ramírez-Mares, Vladimir Sánchez-López, Mario Ramírez-Lepe, Raúl Salas-Coronado, Norma F. Santos-Sánchez, Rogelio Valadez-Blanco, Beatriz Hernández-Carlos

**Affiliations:** ^1^Instituto de Recursos, Universidad del MarPuerto Ángel, México; ^2^Instituto de Biotecnología, Universidad del PapaloapanTuxtepec, México; ^3^Unidad de Investigación y Desarrollo en Alimentos, Instituto Tecnológico de VeracruzVeracruz, México; ^4^Instituto de Agroindustrias, Universidad Tecnológica de la MixtecaHuajuapan de León, México

**Keywords:** filamentous fungi, antifungal activity, plant extracts, *Adenophyllum aurantium*, *Alloispermum integrifolium*, *Ipomoea murucoides*, *Tournefortia densiflora*, *Lantana achyranthifolia*

## Abstract

The antimicrobial activity of 12 plant extracts was tested against the phytopathogens *Alternaria alternata* and *Fusarium solani*. In addition, the compatibility of the extracts toward *Bacillus liqueniformis*, a biofertilizer and a non-target microorganism, was assessed. Plants tested belong to the Euphorbiaceae, Asteraceae, Crassulaceae, Rubiaceae, Convolvulaceae, Verbenaceae, Orchidaceae, Nyctaginaceae, Boraginaceae, and Tiliaceae families and were collected in the State of Oaxaca. The antifungal activity of the plant extracts (50–100 mg/mL) against *A. alternata* and *F. solani*, was determined by measuring the mycelium radial growth and obtaining the minimum inhibitory concentration (MIC) of fungal growth. In addition, with the aim of finding plant extracts which are compatible with a *B. licheniformis* biofertilizer strain and to test the non-toxic nature of the treatments, the toxicity of the extracts toward this strain was evaluated using the agar diffusion method. Azoxystrobin (12 μg) and chloramphenicol (30 μg) were used as positive controls for the pathogens and for the non-target bacteria, respectively. Plant extracts inhibited fungal growth in the ranges of 0.76–56.17% against *F. solani* and 2.02–69.07% against *A. alternata*. The extracts of *Acalypha subviscida*, *Ipomoea murucoides*, *Tournefortia densiflora* and *Lantana achyranthifolia* showed MIC values between 5.77–12.5 mg/mL for at least one of the fungal species. The best treatment, *Adenophyllum aurantium*, exhibited a maximum inhibition for both *F. solani* (56.17%, MIC = 7.78 mg/mL) and *A. alternata* (68.64% MIC = 7.78 mg/mL), and resulted innocuous toward *B. licheniformis*. Therefore, this plant has an outstanding potential for the agroecological control of fungal phytopathogens in industrial crops.

## Introduction

Species of *Alternaria* and *Fusarium* are important plant pathogens which cause significant productivity losses to agriculture worldwide. In Mexico, *A. alternata* and *F. oxysporum* cause black molds in tomato ripe fruits (Gastélum and Gálvez-Figueroa, 2002) and blight of pepper crops (Mojica-Marín et al., 2009), respectively. Nowadays, synthetic fungicides are used indiscriminately for the control of these pathogens which not only damage the environment but also are toxic to the final consumers. Botanical fungicides can be used as a healthy and environmentally friendly alternative to synthetic fungicides. To be used in vegetable production it is desirable that these products are specifically toxic to filamentous fungi without damaging non-target organisms. Soil bacteria, such as *Bacillus* species possess fungicide and biofertilizer activity as they promote plant growth and health, such as *B. subtilis*, *B. pumilus*, *B. amyloliquefaciens*, and *B. licheniformis* (Pérez-García et al., 2011; Dimkić et al., 2013). Therefore, *Bacillus* species can be considered as non-target organisms while assessing new botanical fungicides. On the other hand, some vegetable families have shown fungicide activity against *Fusarium* and *Alternaria* species: Asteraceae (Jasso de Rodríguez et al., 2007), Euphorbiaceae (Gamboa-Angulo et al., 2008; Ascacio-Valdés et al., 2013), and Apocyneaceae (Ferreira-Medeiros et al., 2013). However, there are not published studies that assess the effect of plant fungicides on biofertilizer microorganisms such as *Bacillus* species. Considering this, it is important to search new fungicide plant extracts and to study their compatibility with biofertilizer microorganisms since the use of plant fungicide-biofertilizer treatments represents a better agroecologic alternative than the use of chemical fertilizers and synthetic fungicides. Therefore, in this work, the extracts of 12 plants were tested for their antimicrobial activity against *Fusarium solani*, *Alternaria alternata*, and also for their compatibility with *B. liqueniformis*. The species tested belong to the Euphorbiaceae, Asteraceae, Crassulaceae, Rubiaceae, Convolvulaceae, Verbenaceae, Orchidaceae, Nyctaginaceae, Boraginaceae, and Tiliaceae families and were collected in the State of Oaxaca, Mexico (Table 1).

**Table 1:** **Plants from Oaxaca evaluated against the phytopathogen fungi *Fusarium solani* and *Alternaria alternata***.

**Specie (family)**	**Collection site**	**Voucher number**	**Part plant used**	**Extraction solvent**
*Acalypha cuspidata* Jacq. (Euphorbiaceae)	B	25068	Aerial	MeOH
*Acalypha subviscida* S. Watson var. *lovelanddii* McVaugh (Euphorbiaceae)	A	24007	Aerial	MeOH
*Alloispermum integrifolium* (DC.) H. Rob. (Asteraceae)	A	24024	Aerial	MeOH
*Adenophyllum aurantium* (L.) Strother (Asteraceae)	C	25173	Aerial	MeOH
			Root	MeOH, AcOEt
*Echeveria acutifolia* Lindl. (Crassulaceae)	D	25184	Aerial	MeOH
*Galium mexicanum* Kunth (Rubiaceae)	A	23994	Aerial	MeOH
*Ipomoea murucoides* Roem. & Schult. (Convolvulaceae)	D	25227	Leaves	Ether
*Lantana achyranthifolia Desf.* (Verbenaceae)	D	25185	Aerial	MeOH
*Prosthechea varicosa* (Lindl.) W. E. Higgins (Orchidaceae)	A	24054	Aerial	MeOH
			Bulbs	MeOH
*Salpianthus arenarius Humb. &* Bonpl. (Nyctaginaceae)	C	25084	Aerial	MeOH
*Tournefortia densiflora* M. Martens & Galeotti (Boraginaceae)	C	25221	Aerial	MeOH
			Root	MeOH
*Heliocarpus terebinthinaceus (DC.) Hochr. (Tiliaceae)*	D	25225	Seeds	H_2_O, *n*-Hexane

MeOH, methanol; AcOEt, ethylacetate.

A, San Miguel Suchixtepec, Miahuatlán; B, Candelaria Loxicha, San Pedro Pochutla; C, Chepilme Botanic Garden (Universidad del Mar), San Pedro Pochutla; D, Huajuapan de León; E, Ejutla de Crespo.

## Materials and methods

### Chemicals

All solvents used, methanol, ethyl acetate, ethyl ether and *n*-hexane, were reagent grade (Sigma). Potato dextrose agar (PDA), potato dextrose broth (PDB), potato broth (PB), Mueller Hinton (MH) agar, MH broth (MHB), trypticase soy agar (TSA), trypticase soy broth (TSB), were purchase from Difco (Sparks, MD). Methanol (HPLC grade), dimethyl sulfoxide (DMSO-Hybri-Max), and chloramphenicol were obtained from Sigma Chemical (St. Louis, MO). Azoxystrobin (AMISTAR^®^XTRA) was supplied by Syngenta Crop protection S.A (Macquarie, Australia).

### Biological material

#### Fungal strains

*Fusarium solani* was purchased from the National Collection of microbial and cell cultures of CINVESTAV-IPN (CDBB, Mexico), with a control number CDBB-H-1407.

*Alternaria alternata*: The VSL302 strain was isolated from tomato plant leaves with symptoms of early blight disease and collected from a commercial greenhouse located in Huajuapan de León (Oaxaca, Mexico) in September 2012. The infected tissue was washed with distilled water for 30 s followed by a 1 min immersion in 1% v/v (volume/volume) sodium hypochlorite. Subsequently, the tissue was rinsed three times with distilled water and dried using absorbent paper. Fragments of 0.5 cm were cut and incubated in PDA medium at 25°C for 1 week under 12 h darkness/12 h cool white fluorescent light.

*B. licheniformis* MV1 with accession number Genbank KJ190320 was isolated and identified by Valadez-Blanco and collaborators (submitted, Apr, 2014). The strain was grown and maintained on TSA and TSB media, while MH broth and MH agar were used for the experimental tests.

#### Morphological identification of the fungal isolates

Fungal isolates were grown on SNA plates (Nirenberg, 1976) at 25°C with alternating light/darkness photoperiods (12/12 h) for 7d. Microscopic observations were carried out from preparations mounted in 3% KOH using a bright field and phase contrast Leica DM 300 microscope (Leica Microsystems GmbH Wetzlar, Germany). Morphological characteristics of the colonies, mycelium and conidia of the isolates allowed genera identification using the key of imperfect fungi (Barnett and Hunter, 1998).

#### Molecular identification of the fungal isolate

A fungal isolate (VSL302) was grown on PD plates at 25°C with alternating light/darkness photoperiods (12/12 h) for 3d. The mycelium was placed in a microcentrifuge tube and the genomic DNA was extracted using the commercial DNeasy^®^ Plant Mini Kit following the manufacturer's protocol (Qiagen Inc., CA, USA). The amplification of internal transcribed spacers (ITS1, 5.8S, ITS2 rDNA) and the purification of the PCR products were performed according to the work of Sánchez et al. (2007). Purified PCR products were sequenced by MACROGEN (Maryland, USA). Sequence was aligned and matched against the BLAST nucleotide database (http://blast.ncbi.nlm.nih.gov/Blast.cgi) for the molecular identification of the isolate. Phylogenetic construction was made using the ClustalW2 tool (http://www.ebi.ac.uk/Tools/msa/clustalw2/).

#### Plant material

Twelve plant species were collected in the State of Oaxaca (Mexico) between October 2010 and July 2012. These plants were identified by specialists of the Universidad Nacional Autonoma de Mexico (UNAM) and the Universidad Autonoma de Chapingo (UACh). Plant specimens were deposited in the “Jorge Salas Espinoza” Herbarium of the UACh. Plant parts, scientific name, collection site, voucher number, and extraction solvent are listed in **Table 4**. Plant samples were left to dry at room temperature without sunlight exposure. Samples were subsequently chopped and stored in a cool and dry place before further processing.

#### Preparation of extracts

Extracts were prepared from aerial parts except for those indicated in Table 1, for which the bulbs (B) or the roots (R) were used. Pulverized vegetal material, except *I. murucoides* and *H. terebinthinaceus*, was soaked in 1000 ml of methanol (MeOH) for 2 weeks at room temperature (25°C). Each extract was filtered through a Whatman No. 1 filter paper and the solvent was eliminated using a rotary evaporator at 50°C under reduced pressure. Methanol extract of *A. aurantium* roots was partitioned with AcOEt and the solvent was eliminated.

The dried and ground mixture of the leaves (100 g) of *I. murucoides* was ultrasonic-assisted extracted with 1 L of MeOH acid solution MeOH (990 MeOH + 10 mL AcOH pH 4) in a sonication cleaning bath operated at a frequency of 40 kHz and an ultrasonic input power of 180 W with a useable volume of 6 L (Ultrasonic bath SN-3200 DTN, CE). The extraction was performed by 20 min (twice times) at room temperature. Then, the combined extract was filtered and the solvent evaporated to dryness under reduced pressure. The crude extract was suspended in diluted AcOH (1% v/v, 100 mL at pH 4) and was extracted with Et_2_O (8 × 125 mL). The Et_2_O sub-index extracts were combined, dried over Na_2_SO_4_, filtered and concentrated under reduced pressure to afford an Et_2_O soluble part.

The dried and ground mixture of the seeds (100 g) of *H. terebinthinaceus* was ultrasonic-assisted extracted with 700 mL of aqueous EtOH (90% v/v) in a sonication cleaning bath operated at a frequency of 40 kHz and an ultrasonic input power of 180 W with a useable volume of 6 L (Ultrasonic bath SN-3200 DTN, CE). The extraction was performed by 30 min (three times) at room temperature. Then, the combined extract was filtered and the solvent evaporated to dryness under reduced pressure. The crude extract was suspended in H_2_O (200 mL) and was extracted with hexane (4 × 200 mL). The hexane extracts were combined, dried over Na_2_SO_4_, filtered and concentrated under reduced pressure to afford a hexane soluble part. The aqueous solution was extracted with AcOEt (4 × 200 mL), and was dried over Na_2_SO_4_, filtered and concentrated under reduced pressure. To the remaining MeOH aqueous solution was added MeOH to afford a beige precipitate. The dried and powdered crude extracts were kept at 4°C and protected from light and moisture until further use. Saturated solutions in DMSO (Table 2) of each extract were prepared prior to their use.

**Table 2:** **Percent of inhibitory activity on mycelial radial growth of *F. solani* and *A. alternata* produced by crude plant extracts**.

**Treatment**	**Conc**.	**Fungi strains**	
	**mg/mL**	***F. solani***	***A. alternata***
Azoxystrobin (positive control)	0.060	74.30 ± 5.96	61.17 ± 6.93
*Adenophyllum aurantium*	100	7.40 ± 8.46^*^	9.55 ± 6.79^*^
*Adenophyllum aurantium* R	100	7.34 ± 1.17^*^	2.02 ± 10.16^*^
*Adenophyllum aurantium* R-AcOEt	100	56.17 ± 3.38^*^	68.64 ± 1.97
*Acalypha cuspidata*	50	4.66 ± 3.72^*^	−24.92 ± 2.25^*^
*Acalypha subviscida*	100	16.31 ± 1.68^*^	5.79 ± 9.25^*^
*Alloispermum integrifolium*	100	3.81 ± 3.29^*^	6.09 ± 6.32^*^
*Echeveria acutifolia*	71	17.54 ± 1.37^*^	−38.92 ± 7.28^*^
*Galium mexicanum*	50	10.90 ± 1.66^*^	5.09 ± 4.10^*^
*Lantana achyrantifolia*	100	12.15 ± 0.87^*^	10.77 ± 4.47^*^
*Prostechea varicosa* B	50	−5.25 ± 2.58^*^	−41.44 ± 3.18^*^
*Prostechea varicosa* R	50	4.39 ± 2.11^*^	3.40 ± 3.01^*^
*Salpianthus arenarius*	67	1.05 ± 3.78^*^	−11.86 ± 3.94^*^
*Tournefortia densiflora*	100	0.76 ± 6.06^*^	−22.05 ± 5.78^*^
*Tournefortia densiflora* R	70	52.42 ± 1.96^*^	69.07 ± 2.00
*Heliocarpus terebinthinaceus* S-Aq	100	28.74 ± 1.40^*^	−18.11 ± 3.83^*^
*Heliocarpus terebinthinaceus* S-Hx	200	15.78 ± 3.49^*^	−17.32 ± 8.29^*^
*Ipomoea murucoides* Et_2_O	100	28.19 ± 2.96^*^	−25.45 ± 4.92^*^

B, bulb; R, root; S, seeds. Aerial parts were used for rest of plants.

The extracting solvent is MeOH except when otherwise is indicated. AcOEt, ethylacetate; Aq, aqueous; Hx, hexane; Et_2_O, ethyl ether.

Results are expressed as the mean ± S.D. (n = 3).

Data were analyzed by One-Way ANOVA followed by Dunnett test.

* Significant difference between each treatment and the positive control are shown as p < 0.05.

#### Inhibitory activity on radial growth of fungi

To evaluate the mycelial growth inhibition of the fungal pathogens by the vegetable extracts, *F. solani* and *A. alternata* were cultivated for 5d at 25°C on PDA; the mycelia was harvested and used for the radial growth assays. Following this, 20 μL of each treatment (DMSO, azoxystrobin or plant extract) was put in the center of a PDA plate (60 mm diameter). Azoxystrobin is a commercial synthetic fungicide and was used in this study as the positive control. When the treatments were absorbed into the agar, a 5 mm diameter size plug from the PDA fungal cultures was inoculated on the center of the plate. Each assay was replicated three times. The cultures were incubated for 5d at 28°C and exposed to white light. The radial mycelial growth was determined after 5d by calculating the mean of two perpendicular mycelial-growth diameters for each replicate. The inhibitory activity to radial growth (IR) was calculated according to the following formula (Pinto et al., 1998):

$$\%\text{ IR}=\frac{\text{Dc}-\text{Dt}}{\text{Dc}}$$

where IR = percentage of mycelial growth inhibition, Dc = average diameter of the fungal mycelial-growth of the negative control (DMSO), Dt = average diameter of the fungal mycelial-growth treated with the extracts or the positive control.

According to the availability of extracts and their growth inhibition results, extracts of *A. aurantium*, *A. subviscida*, *G. mexicanum*, and *L. achyranthifolia* were screened at three concentrations for mycelia inhibition of both fungi. Additionally, *A. integrifolium*, and *E. acutifolia* were tested against *F. solani*. Each treatment was diluted in a 1:0, 1:2, and 1:4 ratio and screened for mycelia inhibition following the same methodology described above.

#### Determination of the minimal inhibitory concentration

The MIC was obtained for all the extracts by using a two-fold broth dilution method according to Sasidharan et al. (2012). Sterile PDB medium (300 mL) supplemented with 0.2% Tween 80 (v/v) was inoculated with a pathogenic fungus using 5d cultures grown in 6 mm diameter disks (10). Subsequently, the culture was incubated at 28°C for 7d at 150 rpm with white light exposure. After that, the culture was poured into a sterile Erlenmeyer flask with 100 mL of distilled water supplemented with 0.2% Tween 80 (v/v) and stirred with a magnetic bar for 30 min. The resulting solution, containing the spores, was filtered through a sterile gauze. The spore concentration was adjusted to 10^4^ spores/mL. The treatments were prepared according to the maximum extract concentration used in the antifungal assay plate. 200 μL of the treatment (DMSO, azoxystrobin or plant extract) was mixed with 800 μL of sterile water to get an X treatment concentration (mg/mL); a 0.60 mg/mL concentration was used for the azoxystrobin treatment. Ten sterile test tubes were arranged in a test tube rack and 1 mL of sterile water and 0.2% Tween 80 (v/v) (emulsifying agent) was pipetted into each test tube. Subsequently, a twofold serial dilution of the X treatment concentration was performed. The pathogen inoculum (1 mL, 10^4^ spores/mL) was pipetted into each of the test tubes containing the treatment, thus obtaining the final treatment concentrations: X/4, X/8, X/16, X/32, X/64, X/128, X/256, X/512, X/1024, and X/2048. Finally, the tubes were incubated at 28°C for 7d, in the shaking incubator at 150 rpm and exposed to white light. The MIC value was determined as the lowest concentration of plant extract that completely inhibited the visible growth of the fungal pathogenic strains. Each assay was carried out in triplicate.

#### Compatibility of the plant extracts with B. licheniformis MV1

The effect of the vegetable extracts on *B. licheniformis* MV1 growth was evaluated by the agar diffusion method (Bauer et al., 1966). The strain was cultured on TSA plates at 37°C for 24 h. Following this, 4 mL of MHB was inoculated with the strain and incubated for 2 h at 37°C. This culture was adjusted to the 0.5 MacFarland standard (0.048 M BaCl_2_ 0.5 mL + 0.18 M H_2_SO_4_ 99.5 mL) for the susceptibility test. MHA dishes were impregnated with 150 μL of the adjusted strain suspension using sterile cotton swabs. Chloramphenicol (30 μg) and DMSO (25 μL) were used as positive and negative controls, respectively. Treatment application (25 μL) was performed directly on the solid medium (Table 1). The plates were incubated at 37°C for 24 h, and the diameter of the inhibition zones (I.D.) was measured. All assays were carried out in triplicate.

### Statistical analysis

Data are shown as mean ± SD of three different experiments. Statistical analyses were performed using a One-Way ANOVA, with Dunnett post test. All comparisons were made relative to positive control and significant difference was indicated as ^*^p < 0.05. Growth inhibition results at three concentration levels were compared using Tukey's studentized range (HSD) test, with a *p* = 0.05 using the SAS^®^ v.8.0 software (USA).

## Results

### Identification of the fungal isolate

**Figure 1:**
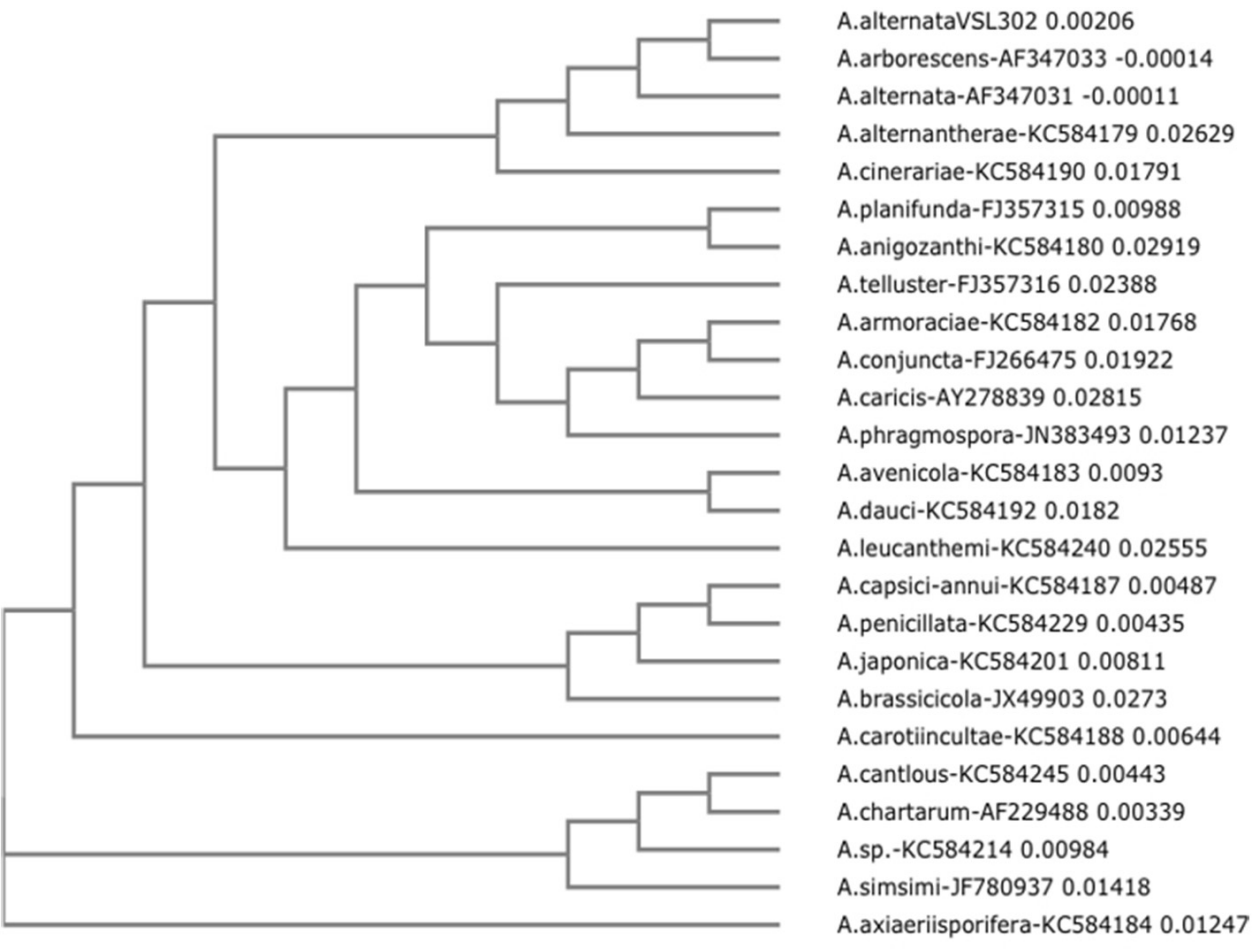
**Neighbor-joining (NJ) tree of ITS1-5.8s-ITS2 from *Alternaria alternata* VSL302**. The tree is based on ITS1-5.8s-ITS2 sequences of 24 strains of *Alternaria*. Each strain correspond to a taxonomic section of the genus *Alternaria*. It is observed that *A. alternata* VSL302 grouped with the strains that correspond to the genus and species *Alternaria alternata* (*A. arborescens* AF347033 and *A. alternata* AF347031). The NJ tree was generated with the PAUP program using the neighbor-joining method. Numbers on branches indicate bootstrap values from an analysis of 1000 replicates.

The mycelium of the fungal colonies grown on synthetic nutrient-poor agar (SNA) plates was greenish gray at the beginning of the incubation and greenish black after 7 days of incubation. Conidia were obclavate and ellipsoidal and had both cross and longitudinal septa. The isolate ribosomal DNA region containing the ITS1-5.8s-ITS2 sequence was amplified from genomic DNA using the primers ITS1 and ITS4. The amplified region showed a 99% homology with *Alternaria alternata* strains (GeneBank KF039678, JX241640, JN673372). A neighbor joining (NJ) tree (Figure 1) was obtained after aligning the ITS1-5.8s-ITS2 sequence of *A. alternata* VSL302 with 24 other GenBank-retrieved sequences, representing known *Alternaria* species from 22 different sections of *Alternaria* (Woudenberg et al., 2013). In particular, *A. alternata* VSL302 grouped with *A. arborescens* AF347033 and *A. alternata* AF347031 that belong to the taxonomic section of Alternata. Thus, based on the phenotype characteristics and by sequence analysis of the ITS gene, the VSL302 isolate was identified as *A. alternata*.

### Inhibitory activity on mycelial radial growth

The results of the mycelial growth assay are listed in Table 2. Concentration of each extract was based on its maximum solubility in DMSO and the amount applied into each plate was based on the minimum volume capable of diffusing into the agar (20 μL). The low extract solubility is due to the fact that all the extracts contained non-polar compounds, except *H. terenbithinaceous* aqueous extract, which could be observed in the solubility tests. Root extracts of *A. aurantium* R-AcOEt (100 mg/mL) and *T. densiflora* R (70 mg/mL) had the maximum mycelial inhibition on *F. solani* (56.17 and 52.42%, respectively) and *A. alternata* (68.64 and 69.07%, respectively). The inhibition of the positive control (0.012 mg/mL) was 74.30 ± 5.96 and 61.17 ± 6.93% for *F. solani* and *A. alternaria*, respectively. Other extracts, such as those from *L. achyranthifolia*, *H. terenbinthinaceus* Ac, *H. terenbinthinaceus* Hx, *I. murucoides* and *A. subviscida*, also presented some antifungal activity (10–28.7%). On the other hand, eight extracts stimulated the mycelial growth of *A. alternata* (−11.86 ± 3.94 to −41.44 ± 3.18%), while one stimulated that of *F. solani* (−5.25 ± 2.58%).

**Figure 2:**
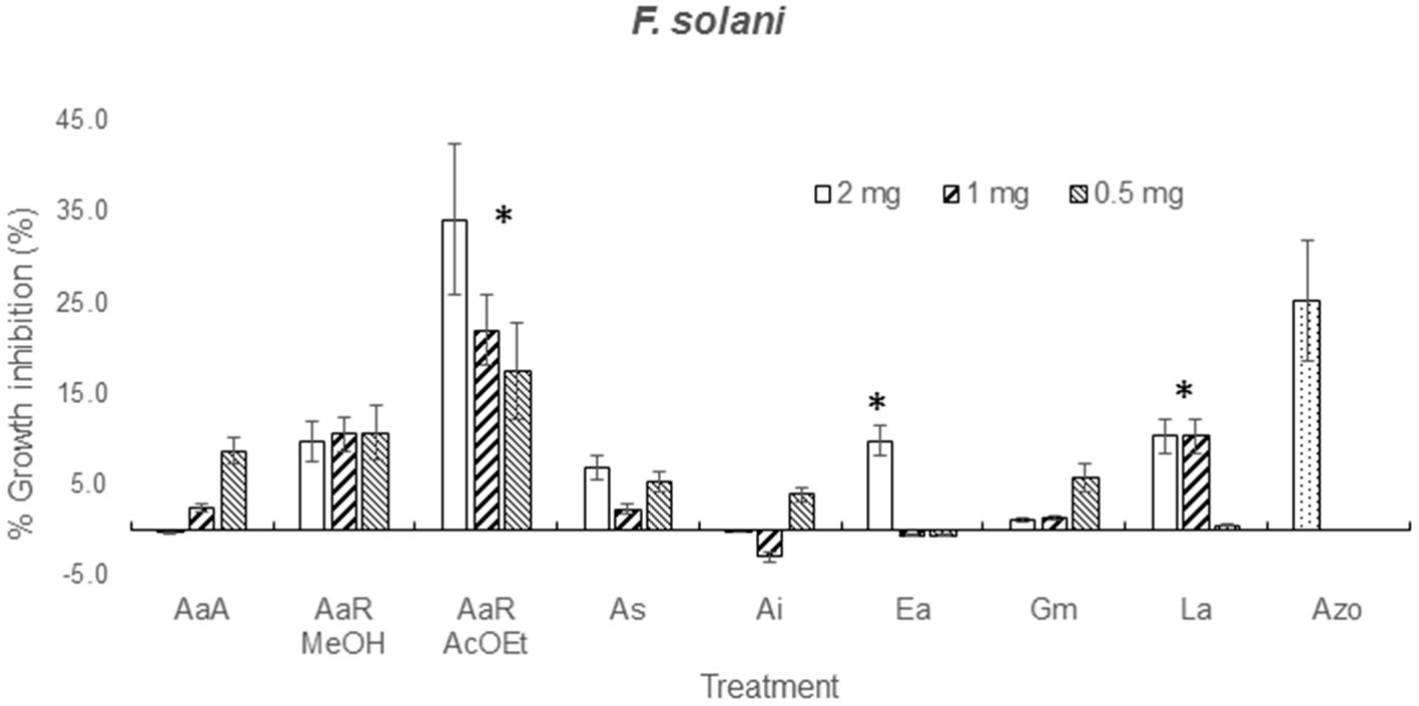
**Zone of inhibition (%) due to methanol extracts of aerial part (AaA) and roots (AaR MeOH and AaR AcOEt) of *A. aurantium*, aerial parts of *A. subviscida* (As), *A. integrifolium* (Ai), *E. acutifolia* (Ea), *G. mexicanum* (Gm), and *L. achyranthifolia* (La) at different concentrations against *F. solani***. Vegetable specie, R = roots and solvent methanol (MeOH) or ethylacetate (AcOEt) are indicated by abbreviations between parenthesis. Different responses observed at three concentrations are marked (^*^) (Tukey; *P* = 0.05). Azo; azoxystrobin (0.012 mg).

**Figure 3:**
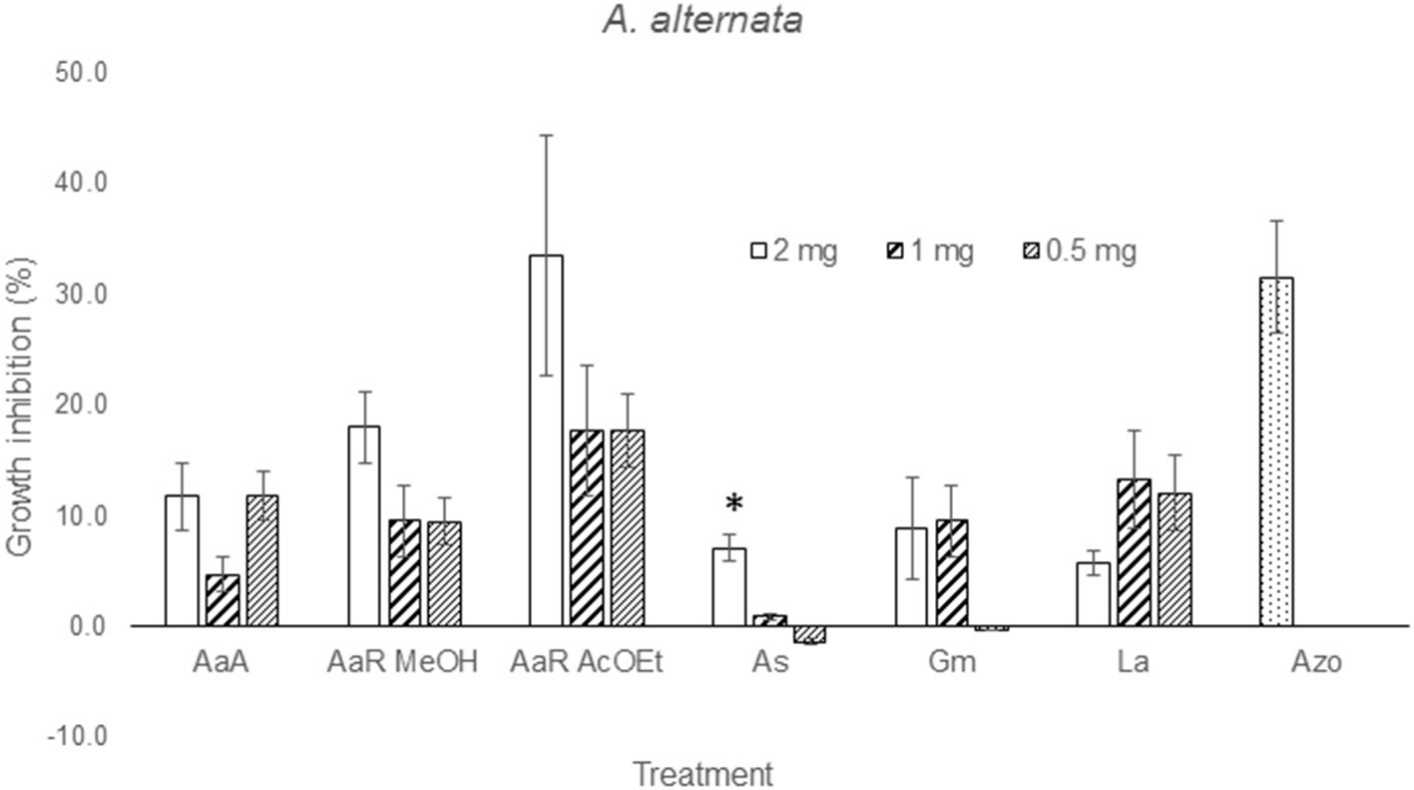
**Zone of inhibition (%) due to methanol extracts of aerial part (AaA) and roots (AaR MeOH and AaR AcOEt) of *A. aurantium*, aerial parts of *A. subviscida* (As), *G. mexicanum* (Gm), and *L. achyranthifolia* (La) at different concentrations against *A. alternata***. Vegetable specie, R = roots and solvent methanol (MeOH) or ethylacetate (AcOEt) are indicated by abbreviations between parenthesis. Different responses observed at three concentrations are marked (^*^) (Tukey; *P* = 0.05). Azo; azoxystrobin (0.012 mg).

The effect of the dilution of the extracts on the activity against *F. solani* probed to be dose-independent, except for *A. aurantium* R AcOEt, *E. acutifolia*, and *L. achyranthifolia* extracts. The activity of *A. aurantium* and *L. achyranthifolia* extracts was reduced when treatments were diluted 1:2 and 1:4 (Figure 2). On the other hand, dose-dependent activity against *A. alternata* was observed only for the *A. subviscida* extract, whose inhibitory effect switched to not inhibition and growth promotion when the treatments were diluted 1:2 and 1:4, respectively (Figure 3). Similar results were observed for the *E. acutifolia* extract activity against *F. solani* (Figure 2).

### Minimal inhibitory concentration

Table 3 shows the minimal inhibitory concentration (MIC) determined as the lowest concentration at which no growth occurs (Sasidharan et al., 2012). *I. murucoides* extract showed the best antifungal activity on *F. solani* (MIC = 5.77 mg/mL), followed by *L. achyranthifolia* on *A. alternata* (MIC = 6.25 mg/mL), *A. aurantium* AcOEt on both strains (MIC = 7.78 mg/mL), and *A. subviscida* on *A. alternata* (MIC = 8.51 mg/mL). The extracts MIC values ranged from 38.5 to 56.8 times the value of the positive control (MIC = 0.15 mg/mL). The rest of the extracts had MIC values from 11.54–50 mg/mL or were unable to inhibit fungal growth.

**Table 3:** **Antifungal activities (MIC) of crude plant extracts of some plants from Oaxaca against *F. solani* and *A. alternata***.

**Treatment**	**MIC (mg/ml)**	
	***F. solani***	***A. alternata***
Azoxystrobin (positive control)	0.15	0.15
*Adenophyllum aurantium*	27.73^*^	27.73^*^
*Adenophyllum aurantium* R	20.00^*^	20.00^*^
*Adenophyllum aurantium* R-AcOEt	7.78^*^	7.78^*^
*Acalypha cuspidata*	12.50^*^	12.50^*^
*Acalypha subviscida*	NEG	8.51^*^
*Alloispermum integrifolium*	33.76^*^	16.88^*^
*Echeveria acutifolia*	17.75^*^	17.75^*^
*Galium mexicanum*	15.00^*^	15.00^*^
*Lantana achiranthifolia*	12.50^*^	6.25^*^
*Prostechea varicosa*-B	NEG	NEG
*Prostechea varicosa*-R	NEG	14.70^*^
*Salpianthus arenarius*	NEG	NEG
*Tournefortia densiflora*	11.75^*^	11.75^*^
*Tournefortia densiflora* R	16.13^*^	16.13^*^
*Heliocarpus terebinthinaceus* S-Ac	NEG	NEG
*Heliocarpus terebinthinaceus* S-Hx	50^*^	50^*^
*Ipomoea murucoides*-Et_2_O	5.77^*^	11.54^*^

B, bulb; R, root; S, Seeds. Aerial parts were used for rest of plants.

The extracting solvent is MeOH except when otherwise is indicated. AcOEt, ethylacetate; Aq, aqueous; Hx, hexane; Et_2_O, ethyl ether.

NEG, Negative (unable to inhibit the fungi growth).

Data were analyzed by One-Way ANOVA followed by Dunnett test.

* Significant difference between each treatment and the positive control are shown as p < 0.05.

### Inhibition of *B. licheniformis* MV1 growth

The *A. aurantium* R-AcOEt extract (Table 4) resulted in a low growth inhibition of the bacteria (1.56 ± 1.81 mm). On the other hand, *T. densiflora* R caused a significant growth inhibition (14.60 ± 1.02 mm) in comparison with the value obtained by the positive control, chloramphenicol (20.93 ± 0.20 mm). Other extracts with low or null inhibitory activities were *H. terenbinthinaceous* Aq (1.56 ± 0.28 mm), *A. integrifolium* (0.00 ± 0.0 mm), *A. aurantium* A (3.48 ± 0.79 mm) and *A. aurantium* R (0.0 ± 0.0 mm).

**Table 4:** **Toxic activity of some plants of Oaxaca against the growth of *B. licheniformis* MV1**.

**Treatment**	**Inhibition zone (mm)**	**Standard deviation**
Chloramphenicol (positive control)	20.93	0.20
*Adenophyllum aurantium*	3.48^*^	0.79
*Adenophyllum aurantium* R	0.00^*^	0.00
*Adenophyllum aurantium* R-AcOEt	1.56^*^	1.81
*Acalypha cuspidata*	10.06^*^	0.55
*Acalypha subviscida*	21.43	0.49
*Alloispermum integrifolium*	0.00^*^	0.00
*Echeveria acutifolia*	14.57^*^	0.61
*Galium mexicanum*	18.02^*^	0.80
*Lantana achyrantifolia*	15.93^*^	0.45
*Prostechea varicosa B*	10.42^*^	1.40
*Prostechea varicosa* R	14.36^*^	1.29
*Salpianthus arenarius*	11.43^*^	1.34
*Tournefortia densiflora*	21.19	1.87
*Tournefortia densiflora* R	14.60^*^	1.02
*Heliocarpus terebinthinaceus* S-Ac	1.56^*^	0.28
*Heliocarpus terebinthinaceus* S-Hx	13.23^*^	1.58
*Ipomoea murucoides* - Et_2_O	13.21^*^	0.72

B, Bulb; R, root; S, seeds. Aerial parts were used for rest of plants.

The extracting solvent is MeOH except when otherwise is indicated. AcOEt, ethylacetate; Aq, aqueous; Hx, hexane; Et_2_O, ethyl ether.

Results are expressed as the mean ± S.D. (n = 3).

Data were analyzed by One-Way ANOVA followed by Dunnett test.

* Significant difference between each treatment and the positive control are shown as p < 0.05.

## Discussion

### Antifungal activity

*F. solani* was the pathogenic strain with more susceptibility to mycelial growth inhibition caused by four extracts (*I. murucoides*, *H. terenbithinaceous*, *T. densiflora* R, and *A. aurantium* R AcOEt), which showed inhibition percentages greater than 20%. MIC values lower than 10 mg/mL were obtained for *F. solani* with two extracts (*A. aurantium*-R AcOEt, and *I. murucoides*). On the other hand, only two extracts displayed inhibition percentages greater than 20% for *A. alternata* (*T. densiflora* R, and *A. aurantium* R AcOEt). However, MIC values lower than 10 mg/mL were obtained for this strain with three extracts (*A. aurantium*-R AcOEt, *A. subviscida* and *L. achyranthifolia*). Therefore, *A. alternata* was the most sensitive strain to sporulation inhibition and *F. solani* was more sensitive to mycelial growth inhibition.

Root extracts of *A. aurantium* AcOEt and *T. densiflora* had the highest mycelial growth inhibition against both pathogenic strains. In Oaxaca, the aerial parts of these vegetable species are used to treat infectious diseases (Alonso-Castro et al., 2011). Osuna et al. (2005) reported that *T. densiflora* leaves had antimicrobial activity against bacteria causing intestinal infections. However, the toxic activity of *Tournefortia* species against filamentous fungi has not been previously reported. At a family level, some Boraginaceae species are toxic toward *Aspergillus niger, A. flavus*, *Rhizoctonia phaseoli* (4 mg/disk; mycelial inhibition >50%) and *R. solani* (IC_50_ 0.180 mg/mL) (Jain et al., 2000; Hernández et al., 2007). Methanol extracts from aerial and root parts of *A. aurantium* were not toxic to any fungal species, but *A. aurantium* ethyl acetate (AcOEt) extracts from roots showed a significant antifungal activity against both fungi strains, with mycelial growth inhibition greater than 50% and a MIC value of 7.78 mg/mL. *Adenophyllum* genus belonging to the subtribe pectidinae is characterized by the presence of thiophenes as 5-(4-hydroxy-1-butenyl)-2-2′-bithienyl, 5-(4 acetoxy-butenyl)-2,2′-bithienyl, and α-terthienyl (Downum et al., 1985). This kind of thiophenes inhibited *in vitro* spore germination and mycelial growth of two *F. oxysporum* strains at concentrations within the μg/mL scale (Kourany and Arnason, 1988). There are reports about the phototoxicity of thiophenes through singlet oxygen or free radicals formation (Evans et al., 1986) which in turn causes damage to the membranes. Arnason et al. (1998) reported this effect in which the thiophene phenylheptatriyne inhibited ^14^C phenylalanine uptake and respiration and enhanced K^+^ leakage in *Fusarium culmorum*. Additionally, a terthiophene derivative led to a phospholipid peroxidation chain and oxidative damage of membrane proteins through a triplet state of the derivative (Saito et al., 2001). Therefore, the mycelial growth inhibition observed by *A. aurantium* roots extracts was probably caused by the disruption of the membrane functions.

The antibacterial activity of some of these extracts has been reported for *I. murucoides* (Corona-Castañeda et al., 2013), *G. mexicanum* (Bolívar et al., 2011) and *L. achyranthifolia* (Hernández et al., 2008). In this study, the extracts of *I. murucoides* and *L. achyranthifolia* had MIC values of 5.77 mg/mL against *F. solani* and 6.25 mg/mL against *A. alternata*. No activity against filamentous fungi has been reported for these species. Some authors have described species of the *Lantana* and *Ipomoea* genus with toxic activity against filamentous fungi: *L. camara* was toxic to *Alternaria* spp. (15 mg/mL, mycelial growth inhibition >50%) (Srivastava and Singh, 2011) and to *Fusarium oxysporum* (MIC = 0.08–2.5 mg/mL) (Mdee et al., 2009), whereas *I. batatas* was toxic to *Rhizopus stolonifer* (EC_50_ = 2.2 g/l) (Stange et al., 2001).

A significant antifungal activity of *H. terebinthinaceous* seed extracts was expected since these extracts contain the flavonoid tiliroside (0.59%) (Santos-Sánchez et al., 2014). This flavonoid was also isolated from *Picea neoveitchii* and showed a mycelial growth inhibition of 55.6 ± 5.1% (100 μg/disk) against *Alternaria mali* (Chen et al., 2012). On the contrary, *H. terebinthinaceous* extracts slightly increased the mycelial growth of *A. alternata* (−18.11 ± 3.83 and −17.32 ± 8.29%, with the Aq and Hx extracts, respectively) (Table 2).

The antifungal activity of *E. acutifolia* and *A. subviscida* has not been studied. These extracts had moderate antifungal activity against *F. solani*. The extract of *E. acutifolia*, a Crassulaceae, showed a value of 17.54 ± 1.37% as mycelial growth inhibition percentage against *F. solani*, which can be considered a weak response. Antifungal species from the Crassulaceae family with antifungal activity are not common, e.g., *Sedum ooxypetalum* (Navarro-García et al., 2003) and *S. acre* (Stanković et al., 2012) which showed toxicity to *Aspergillus niger* (MIC = 8–20 mg/mL). The extract of *Acalypha cuspidata* showed not significant antifungal activity but *A. subviscida* slightly reduced mycelial growth of *F. solani* (16.31 ± 1.68%) and showed one of best MIC values (8.51 mg/mL) against *A. alternata*. Therefore, this species should be considered as a good candidate against filamentous fungi together with other *Acalypha* species reported in the literature with MIC values of 9.5–16.5 mg/mL for *A. hispida* (Ejechi and Souzey, 1999) and 1–4 mg/mL for *A. diversifolia* and *Acalypha* sp. (Niño et al., 2012). Other fungicides belonging to the *Acalypha* genus are *A. gaumeri*, *A. wilkesiana*, and *A. indica* (Alade and Irobi, 1993; Gamboa-Angulo et al., 2008; Devi et al., 2013; Maya and Thippanna, 2013).

The extracts of *S. arenarius* (Nyctaginaceae) and *P. varicosa* (Orchidaceae) showed no toxic significant fungicide activities, except for *P. varicosa* R against *A. alternata* with a MIC value of 14.70 mg/mL. Toxic activity toward filamentous fungi related to Nyctaginaceae species has not been reported, whereas reports for the Orchidaceae species are scarce; e.g., *Nervilia aragoana* (Reddy et al., 2010) and *Cypripedium macronthos* (Shimura et al., 2007).

Essential oils mixtures and extracts from plants with fungicide potential reported elsewhere possess MIC values of 0.03–2.5 mg/mL, which are 4–100 times greater than those of positive controls such as ketoconazole or azoxystrobin (Svetaz et al., 2004; Mdee et al., 2009; Plodpai et al., 2013). The best MIC values obtained in this work are 38.5–56.8 times greater than those of the positive control (azoxystrobin): 6.25, 8.51, 7.78, and 5.77 mg/mL for *L. achyranthifolia*, *A. subviscida*, *A. aurantium* R-AcOEt, and *I. murucoides*, respectively. While mycelia growth inhibition >50% was achieved by *A. aurantium* R-AcOEt and *T. densiflora* R. Species from the Verbenaceae, Euphorbiaceae, Asteraceae, Boraginaceae, and Convolvulaceae families were toxic to at least one of the fungal strains. Our results about toxic activity of *T. densiflora* and *I. murucoides* contribute to the scientific knowledge of plants with fungicide potential from the Boraginaceae or Convolvulaceae families. Previously, this activity was reported for *Heliotropium floridum* (Reyna et al., 1997), *Cordia curassavica* (Hernández et al., 2007), *Ipomoea batatas* (Stange et al., 2001) and *I. carnea*, whose latex possesses a quitinase (Patel et al., 2010). Whereas, examples of plants belonging to Euphorbiaceae (Niño et al., 2012; Ascacio-Valdés et al., 2013), Asteraceae and Verbenaceae (Hernández et al., 2008; Díaz-Dellavalle et al., 2011; Pupo-Blanco et al., 2011) with toxic activity on phytopathogens fungi are more common, particularly those from the Asteraceae family (Gamboa-Angulo et al., 2008; Nogueira et al., 2010; Carvalho et al., 2011).

### Mycelial growth inhibition of treatments at several concentrations

From solubility tests, it was observed that all the extracts possessed a mixture of non-polar and polar constituents. Polar and non-polar mixtures were extracted with methanol, non-polar compounds were extracted using ether, *n*-hexanes (Hx) and ethyl acetate (AcOEt), and water was used for the extraction of polar components from *H. terenbithinaceus*. Presence of non-polar compounds in the extracts limited their diffusion into the agar, which in turn caused that the activities of most of the extracts were dose-independent (Figures 2, 3). However, *A. aurantium* R AcOEt, *E. acutifolia*, and *L. achyranthifolia* extracts showed dose-dependent activity against *F. solani*. The activity of *A. aurantium* and *L. achyranthifolia* extracts was reduced when the treatments were diluted in a 1:2 and 1:4 ratio (Figure 2). The effect of dilution of the *A. subviscida* extract on *A. alternata* was similar to that of *E. acutifolia* (Figure 3), whose inhibitory effect shifted to not inhibition and growth promotion when the treatments were diluted. This biphasic dose response was observed because growth stimulation happened at low doses and growth inhibition at high doses. This phenomenon is known as hormesis (Garzon and Flores, 2013) and has been described for some filamentous fungi as *Fusarium oxysporum*, *Pythium aphanidermatum*, and *Penicillium expansum* (Flores and Garzon, 2013) in mycelial growth tests.

### Inhibitory activity against *B. licheniformis*

In the current agroecologic practice, *Bacillus* strains are used to aid organic fertilizers and to control phytopathogens in commercial crops (Pérez-García et al., 2011), as well as to reduce the use of synthetic agrochemicals. Examples of such practices are the control of *Ralstonia solanacearum* in tomato crops by a *Bacillus* sp. strain (Wei et al., 2011) and the combination of *Bacillus subtilis* and *Pseudomonas fluorescens* (biocontrol agents) with extracts of *Allium* spp. to inhibit the mycelial growth of *Alternaria solani* (Latha et al., 2009). With the aim of finding an effective combination of a plant-derived fungicide and a biofertilizer bacteria, such as *B. licheniformis*, the toxicity of 12 plant extracts was tested against this strain. The extracts of *A. aurantium*, *A. integrifolium*, and *H. terenbinthinaceus* Aq did not have a significantly toxic activity against *B. licheniformis*. From these extracts, the antifungal treatments with greater potential are *A. aurantium* AcOEt since it was very active against mycelial growth of *F. solani* and *A. alternata*. In addition, *A. integrifolium*, *A. aurantium* R, and *A. aurantium* R-AcOEt had the greatest toxic activities against spores of *A. alternaria* with MIC values of 16.9, 20.0, and 7.8 mg/mL, respectively.

## Conclusions

The results suggest that among the plants studied, the most promising treatments for use in combination with *Bacillus licheniformis* are the *A. aurantium* roots AcOEt and *A. integrifolium* MeOH extracts. These extracts reduced the mycelial growth and sporulation of both pathogenic fungal strains: *Fusarium solani* and *Alternaria alternata*. In addition, the extracts of these plants did not affect the growth of the biofertilizer *B. licheniformis* and therefore possess an outstanding potential for the agroecologic control of fungal phytopathogens in industrial crops. Other extracts were toxic to *B. liqueniformis* but had antifungal activity against *A. alternata* (*A. subviscida* and *L. achyranthifolia*) and *F. solani* (*I. murucoides*). *T. densiflora* R had antifungal activity against both fungal strains. Therefore, these extracts can be used for the control of phytopathogen fungi in post-harvest foods as papaya (Bautista-Baños et al., 2013) and tomato (Feng et al., 2011). Further studies are needed to determine the bioactive compounds responsible for the antifungal activity of the extracts of *A. aurantium*, *T. densiflora*, *A. subviscida*, *L. achyranthifolia*, and *I. murucoides.*

### Conflict of interest statement

The authors declare that the research was conducted in the absence of any commercial or financial relationships that could be construed as a potential conflict of interest.
